# Detecting clusters of transcription factors based on a nonhomogeneous poisson process model

**DOI:** 10.1186/s12859-022-05090-2

**Published:** 2022-12-09

**Authors:** Xiaowei Wu, Shicheng Liu, Guanying Liang

**Affiliations:** 1 Department of Statistics, Virginia Tech, 250 Drillfield Drive, Blacksburg, VA 24061 USA; 2 Department of Mathematics, Virginia Tech, 225 Stanger Street, Blacksburg, VA 24061 USA

**Keywords:** Transcription factor, Binding site, Nonhomogeneous Poisson process

## Abstract

**Background:**

Rapidly growing genome-wide ChIP-seq data have provided unprecedented opportunities to explore transcription factor (TF) binding under various cellular conditions. Despite the rich resources, development of analytical methods for studying the interaction among TFs in gene regulation still lags behind.

**Results:**

In order to address cooperative TF binding and detect TF clusters with coordinative functions, we have developed novel computational methods based on clustering the sample paths of nonhomogeneous Poisson processes. Simulation studies demonstrated the capability of these methods to accurately detect TF clusters and uncover the hierarchy of TF interactions. A further application to the multiple-TF ChIP-seq data in mouse embryonic stem cells (ESCs) showed that our methods identified the cluster of core ESC regulators reported in the literature and provided new insights on functional implications of transcrisptional regulatory modules.

**Conclusions:**

Effective analytical tools are essential for studying protein-DNA relations. Information derived from this research will help us better understand the orchestration of transcription factors in gene regulation processes.

**Supplementary Information:**

The online version contains supplementary material available at 10.1186/s12859-022-05090-2.

## Background

Transcription factors (TFs) are the central regulators of gene expression. They are proteins that bind to DNA sequences. By promoting or blocking the recruitment of RNA polymerase to specific genes, TFs control the transcription rate, leading to a measurable downstream effect on gene expression regulation [1]. Through chromatin immunoprecipitation combined with sequencing (ChIP-seq) experiments, it has been found that TF binding sites (TFBSs) on the genome are not randomly distributed but rather cluster together at enhancer or promoter regions [2, 3]. Studying the complex binding patterns among TFs brings to light the quantitative nature of “how TFs work together and interact with each other to regulate gene expression” hence helps detect or predict new regulatory signals [4].

The analysis of TF binding comprises different but related perspectives. For example, one may be interested in the DNA sequences required for regulating gene expression, called *cis*-regulatory modules or CRMs [5–7], on which TFs exhibit co-localized TFBSs. From another point of view, it is also biologically critical to explore combinatorial protein-protein interactions among TFs [8, 9], either direct or indirect, as they indicate co-regulatory mechanisms. A common TF–TF interaction is represented by their cooperative binding to CRMs, in which case the pairs of TFBSs show short distance preferences, suggesting that TFs function coordinatively as a module. Considerable efforts have been devoted to address the issue of cooperative TF binding. Notable works include clustering based on Pearson correlation between co-localization vectors of two TFs [10], identifying context dependent co-regulators from co-bound regions of two TFs [11], testing TF pairs for several distance ranges and orientations [12], detecting TF complexes by enrichment of motif spacings [13], inferring active TF modules by a Gibbs sampler under Bayesian framework [14], etc. Despite the progress, development of computational methods for detecting TF clusters is still insufficient. Existing methods often rely on simplified model assumptions, subjectively selected thresholding parameters, and routine analytic procedures. For example, Chen et al. used a binding site distance threshold of 100 bp to define multiple TF binding loci [10], Cha and Zhou made an assumption that the conditional density for a particular TF is identical across all upstream regions [15], which may be unrealistic in real data, Kazemian et al. identified inter-site spacing bias for a fixed range between 0 and 100 bp then implemented a Fisher’s exact test on the contingency table of site pair counts within or outside the range [12]. Such *ad hoc* or over-simplified settings could cause loss of important information in data and lead to biased interpretations. There is a pressing need for new statistical methods addressing the cooperative binding of TFs in ChIP-seq data.

In this study, we model TF binding events by a nonhomogeneous Poisson process, and develop innovative and flexible statistical methods for clustering multiple-TF binding patterns on a target genomic region. The Poisson process is often used to describe recurrent events randomly located along time or space, and nonhomogeneous Poisson processes relax the the Poisson process assumption by allowing the arrival rate to vary, thereby serving as a more flexible and faithful model in many applications. We model the occurrence of TFBSs on the genome by a nonhomogeneous Poisson process because of three reasons. First, the TF binding events in non-overlapping intervals are naturally independent. Second, within each tiny interval on the genome, the binding of TF is a rare event. Last, TFs tend to bind more frequently to particular regions, e.g., around the transcription start site, which can be properly characterized by the intensity function of the nonhomogeneous Poisson process. Our clustering methods are elicited from the classical paradigm of partitional and hierarchical clustering, with suitable adaptions by incorporating the nonhomogeneous Poisson process likelihood. We demonstrate the outperformance of our methods over traditional window-based clustering by simulation studies, and further apply them to analyze multiple-TF ChIP-seq data in mouse embryonic stem cells (ESCs). This research advances the existing knowledge of clustering analysis on point processes, and provides us new insights on the concerted action of multiple TFs in transcriptional regulation.

## Methods

### Modeling TF binding events by NHPP

Nonhomogeneous Poisson processes (NHPPs) are commonly used to model the occurrence of recurrent events along time or space. NHPPs assume that events occurring in any tiny interval $[t, t+\Delta t], \Delta t\rightarrow 0$ follow independent Bernoulli distribution with success probability determined by an intensity $\lambda (t)$. A wide range of recurrent events, from the arrival of customers at a restaurant to the stream of photons from an optical modulator, are seen to satisfy such an assumption thus can be modeled by NHPPs. Mathematically, an NHPP is a counting process $\{N(t), t\ge 0\}$ with intensity function $\lambda (t), t\ge 0$ if

(i) $$N(0)=0$$,
(ii) $\{N(t), t\ge 0\}$ has independent increments,
(iii) $$P\{N(t+\Delta t)-N(t)\ge 2\}=o(\Delta t)$$,
(iv) $$P\{N(t+\Delta t)-N(t)=1\}=\lambda (t)\Delta t+o(\Delta t)$$

It can be shown that, the number of events occurred in an interval $[t, t+s]$ follows Poisson distribution, that is

$$\begin{aligned} P\{N(t+s)-N(t)=n\}=e^{-(m(t+s)-m(t))}\frac{\left[ m(t+s)-m(t)\right] ^n}{n!}, \end{aligned}$$

where $$m(t)=\int _0^t\lambda (s)ds$$.

In this study, we use NHPPs to model the events of transcription factors binding to the DNA sequence. We assume that the binding pattern of each TF is characterized by an NHPP with a specific intensity function, thus the TF binding site locations are the observed arrival times in the sample paths of the NHPP. Denote the entire genome by a bounded interval $\Omega \subset \mathcal {R}$, and consider a genomic region of interest $D\subset \Omega$ on which a total of *n* TFs have binding sites. The binding site locations of the *i*th TF, $1\le i\le n$ are denoted by $\varvec{s}_i=\{s_{ij}\}, s_{ij}\in D, 1\le j\le n_i$. It is known that, the likelihood of observing $\varvec{s}_i$ in an NHPP with intensity function $\lambda (t)$

1 $$\begin{aligned} \pi (\varvec{s}_i|\lambda (t))\propto \exp \left\{ -\int _D\lambda (s)ds\right\} \prod _{j=1}^{n_i}\lambda (s_{ij}). \end{aligned}$$

We note that, the above model is based on a flexible setting on the genomic region *D*. *D* can be any region of (biological) interest, regardless of its size, large or small. For example, the genomic region *D* can be a certain chromatin state [16], or the upstream region of a gene relative to its transcription start site (TSS) [15].

### NHPP-likelihood-based clustering

The purpose of this study is to develop novel and effective statistical methods for detecting the clustering patterns of transcription factors based on their binding site locations on the genome. Classical clustering methods, including both hierarchical clustering and partitional clustering, may not be directly applicable in this context as they are developed based on distance metrics of multivariate, vector-type data. We therefore propose new, NHPP-likelihood-based clustering methods that are generally suitable for ChIP-seq and similar data and are not necessarily of vector type.

First, we consider partitional clustering of TFBS data by generalizing the K-means algorithm (specifically, the Lloyd algorithm) using NHPP likelihood (1). The idea is still to iteratively reallocate the data to clusters until some criterion is optimized, however, in order to cope with the TFBS data, we make two major adaptions to the newly proposed method:

1. The NHPP intensity function of each cluster plays, conceptually, the role of the centroid of the cluster, therefore estimation of the cluster intensity calibrates the cluster centroid;
2. The distance from the cluster centroid to each sample (i.e., NHPP sample path) is replaced by the negative log-likelihood of observing the sample given the corresponding NHPP intensity function.

Details of this method are provided in Algorithm 1, including two steps: initialization and iteration. In the initialization step, samples are randomly assigned to *k* clusters, and in the iteration step, each sample will be re-assigned to the cluster with largest NHPP likelihood (i.e., to the closest centroid), and then the NHPP intensity functions for the updated clusters will be re-estimated (analogous to re-calculating the cluster centroids). Such iterations keep running until convergence is reached according to the following optimization criterion:

2 $$\begin{aligned} \max _{\varvec{G}} \sum _{i=1}^k \sum _{\varvec{s}_j\in G_i} \log \pi (\varvec{s}_j|\hat{\lambda }_i(t)), \end{aligned}$$

where $\varvec{G}$ denotes the partition of the samples, $G_i$ denotes the *i*th group, and $\hat{\lambda }_i(t)$ is the estimated intensity function of the *i*th group. In fact, such an adapted K-means method is generally applicable to data from any distribution with an explicit likelihood. Some special cases of the likelihood-based K-means include the traditional Euclidean-distance-based K-means where data are from independent homoscedastic normal distribution, and the Mahalanobis-distance-based K-means where data are from independent multivariate normal distribution with common covariance. To see this, considering a simple clustering problem where data *x* in the *i*th cluster are from i.i.d. normal with a known common variance and unknown mean $\mu _i$, the optimization criterion of the likelihood-based K-means can be written as $\max _{\varvec{G}} \sum _{i=1}^k \sum _{x\in G_i} \log \pi (x|\hat{\mu }_i)$, which is equivalent to the distance criterion of the traditional K-means $\min _{\varvec{G}} \sum _{i=1}^k \sum _{x\in G_i} ||x-\hat{\mu }_i||^2$. The adapted K-means method will be called hereafter the **N**HPP-**L**ikelihood-based **K**-means, abbreviated as **NLK**, and similarly in later sections, the adapted hierarchical clustering method will be called the **N**HPP-**L**ikelihood-based **H**ierarchical-clustering, abbreviated as **NLH**.
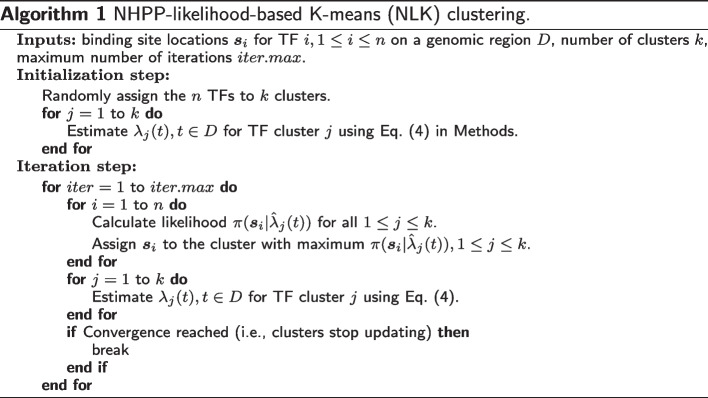


Next, we extend hierarchical clustering by exploiting the NHPP likelihood. For hierarchical clustering, the linkage rule, i.e., the distance between two clusters, plays the most important role. Here, we define a new, NHPP-likelihood linkage

3 $$\begin{aligned} D(G_i, G_j)=-\left[ \sum _{\varvec{s}_k\in G_j} \log \pi (\varvec{s}_k|\hat{\lambda }_i(t))+\sum _{\varvec{s}_k\in G_i} \log \pi (\varvec{s}_k|\hat{\lambda }_j(t))\right] , \end{aligned}$$

so that the traditional hierarchical clustering can be applicable to the TFBS data. This NHPP-likelihood-based hierarchical (NLH) clustering algorithm (bottom-up, or agglomerative) is provided in Algorithm 2.
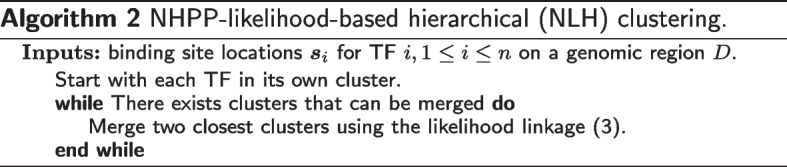


It is worth noting that, NLK and NLH inherited features respectively from K-means and hierarchical clustering. Generally speaking, each of them has its own advantages and disadvantages. For example, NLK usually produces tighter clusters and runs computationally faster, but the optimal number of clusters, *k*, relies on heuristic justification (e.g., by the elbow method [17]). On the other hand, NLH produces more informative clustering results as a hierarchy, however the procedure is sensitive to outliers, and the decision at each step is local optimal rather than global optimal.

### Maximum likelihood estimation of NHPP intensity function using basis expansion

The key of the above NHPP-likelihood-based clustering algorithms lies in the estimation of the NHPP intensity function. This step appears in model identification in Algorithm 1 (i.e., calculating the centroids, or the M-step in the EM algorithm), and in implementing the likelihood linkage in Algorithm 2. In this study, we propose to estimate the NHPP intensity function by maximum likelihood using basis expansion.

Given a cluster of *n* TFs with binding sites independently located on a genomic region *D* and denoted by $\{\varvec{s}_1, \varvec{s}_2, \cdots , \varvec{s}_n\}$, suppose the underlying intensity function is $\lambda (t)$, then by (1), the joint likelihood can be written as

$$\begin{aligned} \prod _{i=1}^n \pi (\varvec{s}_i|\lambda (t))=\exp \left\{ -n\int _D\lambda (s)ds\right\} \prod _{i=1}^n \prod _{j=1}^{n_i}\lambda (s_{ij}). \end{aligned}$$

Now decompose $\lambda (t)$ through signal representation by orthonormal basis, for example by the discrete cosine transform (DCT), so that

$$\begin{aligned} \lambda (t)\sim \sum _{i=0}^\infty c_i\psi _i(t). \end{aligned}$$

Here $\psi _i(t)$ denotes the *i*th orthonormal basis, and $c_i$ is the corresponding coefficient. In particular, we consider the sparse representation case where most coefficients are zeros so that $\lambda (t)$ can be fully characterized by only a few “important” coefficients, denoted by vector $\varvec{c}$. Under this setting, the estimation of $\lambda (t)$ is turned into the estimation of such coefficients and can be achieved via maximum likelihood by numerically searching over the coefficient space,

4 $$\begin{aligned} \hat{\varvec{c}}={{\,\mathrm{arg\,max}\,}}\prod _{i=1}^n \pi (\varvec{s}_i|\lambda (t)). \end{aligned}$$

## Results

### Simulation study to evaluate NLK clustering

We first conduct simulations to evaluate the performance of the proposed NLK clustering method. The simulated data contain binding site locations of multiple TFs on a hypothetical genomic region [*a*, *b*], e.g., [0, 10]. We consider a total of *n* TFs which, according to their binding patterns, can be grouped into *k* clusters. Properly speaking, the binding pattern of each TF is characterized by the intensity function of an NHPP, and TFs in each cluster share the same binding pattern (i.e., intensity function), distinct from those in other clusters.

Depending on the number of TFs *n*, the number of clusters *k*, and the strategy of generating intensity functions for the TF clusters, we explore different scenarios in the simulations. Table 1 lists three scenarios, which may be roughly categorized as “easy”, “moderate”, and “hard” in terms of the clustering difficulty. In both Scenarios 1 and 2, we consider $k=3$ clusters formed by $n=30$ TFs. Scenario 1 adopts a balanced design with the three cluster sizes fixed to 10, 9, and 11, respectively. The binding site locations of the TFs in these clusters are simulated from an NHPP, with the following intensity functions

$$\begin{gathered} {\text{Cluster 1}}:\lambda _{1} (t) = 3\cos \left( {\frac{{2\pi }}{{10}}t} \right) + 3, \hfill \\ {\text{Cluster 2}}:\lambda _{2} (t) = 3\cos \left( {\frac{{3\pi }}{{10}}t} \right) + 3, \hfill \\ {\text{Cluster 3}}:\lambda _{3} (t) = 3\cos \left( {\frac{{4\pi }}{{10}}t} \right) + 3,\quad t \in [0,10]. \hfill \\ \end{gathered}$$

Scenario 2 adopts an unbalanced design with the three cluster sizes fixed to 15, 10, and 5, respectively. Similarly, the binding site locations of the TFs in these clusters are simulated using the following intensity functions

$$\begin{gathered} {\text{Cluster 1}}:\lambda _{1} (t) = 2\cos \left( {\frac{{2\pi }}{{10}}t} \right) - \cos \left( {\frac{{3\pi }}{{10}}t} \right), \hfill \\ {\text{Cluster 2}}:\lambda _{2} (t) = 2\cos \left( {\frac{{3\pi }}{{10}}t} \right) + \cos \left( {\frac{{4\pi }}{{10}}t} \right), \hfill \\ {\text{Cluster 3}}:\lambda _{3} (t) = \cos \left( {\frac{{2\pi }}{{10}}t} \right) - 2\cos \left( {\frac{{4\pi }}{{10}}t} \right),\quad t \in [0,10]. \hfill \\ \end{gathered}$$

**Fig. 1:**
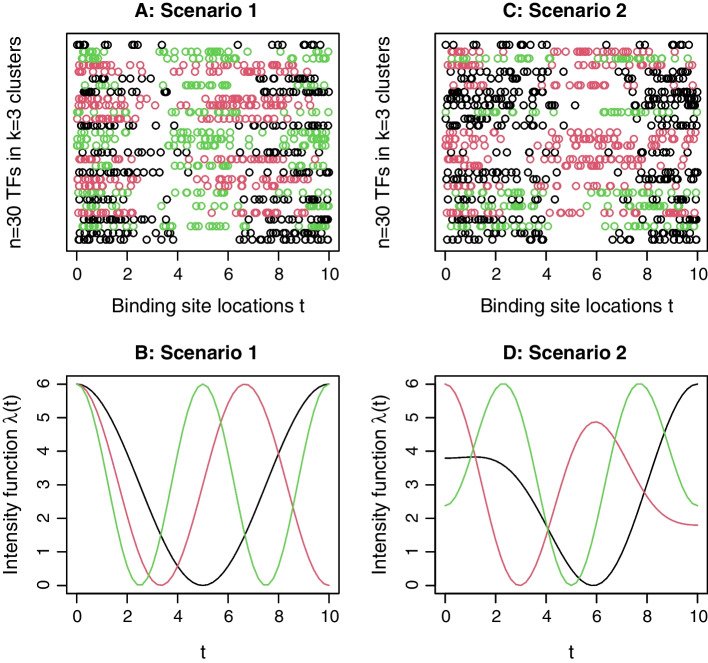
**Demonstration of simulated data in Scenarios 1 and 2 for NLK clustering.** **A**: Binding site locations of the 30 TFs in Scenario 1; **B**: Intensity functions in the three clusters in Scenario 1; **C**: Binding site locations of the 30 TFs in Scenario 2; **D**: Intensity functions in the three clusters in Scenario 2. The three clusters are displayed in black, red, and green colors, respectively

Note that, for consistency, the magnitudes of these intensity functions in Scenario 2 are scaled to [0, 6]. Scenario 3 considers more TFs ($n=100$), more clusters ($k=10$), randomly assigned cluster sizes (random in each simulation), and uses a more flexible design (B-spline basis with degree 6) for the intensity functions. To demonstrate the simulated binding site locations as well as the distinct NHPP intensity functions in the three clusters, we show in Fig. 1 two examples for Scenario 1 (in Fig. 1A and B) and Scenario 2 (in Fig. 1C and D), respectively. An example of the crowded data in Scenario 3 is provided in Additional file 1: Figure S1 for the sake of conciseness of the main text.

**Table 1:** Scenarios used in simulations for NLK clustering

Scenario	No. of TFs *n*	No. of clusters *k*	Cluster size	Intensity function in each cluster
1	30	3	Balanced: (10, 9, 11)	Generated by cosine basis with 1 frequency
2	30	3	Unbalanced: (15, 10, 5)	Generated by cosine basis with 2 frequencies
3	100	10	Randomly assigned	Generated by B-spline basis with degree 6

We apply NLK clustering to the simulated data in the above three scenarios to predict the cluster ID of each TF, and compare it with the true cluster ID to evaluate the clustering performance. Two evaluation criteria are considered, namely the average misclassification rate (AMCR) and the proportion of perfect classification (PPC). The simulation procedure is repeated 1,000 times and in each iteration the misclassification rate is calculated by counting the number of TFs that are classified into the wrong cluster. We note that, the calculation of the misclassification rate may encounter problem due to labelling ambiguity in unsupervised learning. Taking a dataset of six TFs with true cluster IDs [1, 1, 1, 2, 2, 3] as an example, if the clustering yields no error however the predicted cluster IDs are labelled differently as [3, 3, 3, 1, 1, 2], the misclassification calculation will be messed up. To solve this issue, we calculate the misclassification rate by comparing two adjacency matrices (the upper triangular part), one generated from the true clusters, and the other from the predicted clusters. Each (*i*, *j*)th component of the adjacency matrix takes binary values with 1 indicating that the *i*th and *j*th TFs belong to the same cluster, and 0 otherwise. For example, the adjacency matrix in the above example can be seen as

$$\begin{aligned} \left( \begin{array}{cccccc} \cdot &{}1&{}1&{}0&{}0&{}0 \\ &{}\cdot &{}1&{}0&{}0&{}0 \\ &{} &{}\cdot &{}0&{}0&{}0 \\ &{} &{} &{}\cdot &{}1&{}0 \\ &{} &{} &{} &{}\cdot &{}0 \\ &{} &{} &{} &{} &{}\cdot \end{array}\right) , \end{aligned}$$

and is invariant to labelling of the clusters. Denote the true and predicted adjacency matrices (with size $n\times n$) in the *i*th simulation, $1\le i\le T$, by $A_i$ and $\hat{A}_i$, respectively. The average misclassification rate is then defined by

$$\begin{aligned} \text {AMCR}=\frac{1}{T}\sum _{i=1}^T\left\{ \frac{1}{\left( {\begin{array}{c}n\\ 2\end{array}}\right) } \sum \left[ tri(A_i)\ne tri(\hat{A}_i)\right] \right\} , \end{aligned}$$

where *tri*(*A*) denotes a vector containing the upper triangular components of matrix *A*. The PPC is calculated by counting the occurrence of “perfect classification”, i.e., simulation with zero misclassification rate, across the 1,000 iterations. That is,

$$\begin{aligned} \text {PPC}=\frac{1}{T}\sum _{i=1}^T 1_{\left\{ \sum \left[ tri(A_i)\ne tri(\hat{A}_i)\right] =0\right\} }, \end{aligned}$$

where the indicator function $1_{E}=1$ if event *E* happens and 0 otherwise. We choose these two evaluation criteria because the AMCR measures the average accuracy of cluster ID prediction whereas the PPC focuses on the capacity of achieving high quality predictions. Both criteria evaluate the clustering performance, but in different aspects.

Table 2 reports the AMCR and PPC (listed in parentheses) results of NLK clustering using simulated data in Scenarios $1\sim 3$. From this table we see that, NLK clustering achieves very low AMCR in all three scenarios, and obviously the AMCR ascends as the clustering difficulty increases from “easy”, “moderate”, to “hard”. On the other hand, the PPC descends as the clustering difficulty increases. In particular, for Scenario 1, we investigate the relation between the NLK clustering performance and the number of TFs included. The AMCR and PPC results for 10 varying sample sizes $n=15, 30, \cdots , 150$ are included in Additional file 1: Figure S2, showing a decreasing trend of AMCR (also PPC but with a much slower decay rate) as the number of TFs increases.

**Table 2:** Performance of NLK clustering and four other methods using simulated data in Scenarios 1$\sim$3

Methods	AMCR$^*$			PPC$^*$		
	Scenario 1	Scenario 2	Scenario 3	Scenario 1	Scenario 2	Scenario 3
NLK clustering	**0.0044**	**0.0446**	**0.0798**	**96.8%**	**51.1%**	0%
Window-based K-means$^\dag$	0.0294	0.1006	0.1067	68.9%	21.8%	0%
Window-based Hclust$^\dag$	0.0380	0.1222	0.1160	54.2%	11.4%	0%
K-function-based Hclust$^\ddag$	0.0763	0.2022	0.1533	43.1%	0.70%	0%
Co-localization-vector-based Hclust$^\ddag$	0.0220	0.1184	0.1084	68.2%	11.7%	0%

$^*$ AMCR: average misclassification rate; PPC: proportion of perfect classification. The boldface number shows the best result across different clustering methods.

$^\dag$: For these methods, the AMCR and PPC are calculated under the optimal window width such that the AMCR is minimized.

$^\ddag$: For these methods, the multiple TF binding loci are defined by peaks within a prespecified distance threshold, and the AMCR and PPC are calculated under the optimal distance threshold such that the AMCR is minimized

For comparison purpose, Table 2 also lists the clustering results of four other clustering methods: the window-based K-means, the window-based hierarchical clustering, the k-function-based hierarchical clustering, and the co-localization-vector-based hierarchical clustering. The two window-based clustering methods first segment the genomic region into equal-width windows, then calculate for each TF the number of binding sites within each window to convert the TF binding pattern into a vector of counts with unified length, and finally implement the traditional K-means and hierarchical clustering algorithms to the vector of counts. In all three scenarios, we vary the number of windows from 2 to 100, calculate the AMCR and PPC under each window width, and then find the optimal window width that yields the minimum AMCR, and the corresponding PPC. The k-function-based hierarchical clustering is adapted from Cha and Zhou [15] by counting for every two TFs the pairs of binding sites within a prespecified distance threshold and based on which performing hierarchical clustering. The co-localization-vector-based hierarchical clustering [10] computes the Pearson correlation coefficient for every two TFs based on the pair of co-localization vectors and uses it as a similarity measure for hierarchical clustering. In all three scenarios, we set the distance threshold for both the k-function-based and co-localization-vector-based hierarchical clustering to be proportional to the length of the entire genomic region with a ratio varying at $[0.005, 0.01, \ldots , 0.05]$. As shown in Table 2, NLK clearly outperforms the four competitors in all three scenarios, in terms of both AMCR and PPC. More details of the comparison between the two partitional clustering methods, NLK and the window-based K-means, can be found in Additional file 1: Figure S3, where the AMCR of window-based K-means under each window width is shown for the three scenarios, in contrast to the AMCR of NLK. As expected, the AMCR of window-based K-means shows a “V”-shape where the minimum is reached when the optimal window width is chosen.

**Fig. 2:**
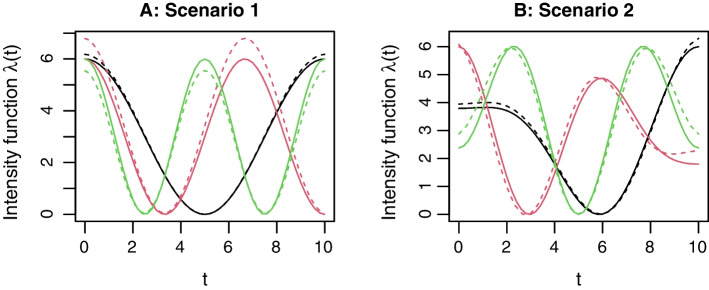
**Demonstration of intensity function estimation in NLK clustering using example data in Fig.** 1. **A**: The true and estimated intensity functions in the three clusters in Scenario 1; **B**: The true and estimated intensity functions in the three clusters in Scenario 2. The true and estimated intensity functions are shown in solid and dashed lines, respectively. The three clusters are displayed in black, red, and green colors, respectively

As a byproduct, the intensity functions in different clusters can be estimated simultaneously. Figure 2 shows the comparison between the estimated (dashed lines) and true (solid lines) intensity functions for the examples provided in Fig. 1. For these two examples from Scenarios 1 and 2, the AMCR is 0, and the estimated intensity functions match the true intensity functions well. For the example from Scenario 3 (corresponding to Figure S1), the intensity estimation results are provided in Additional file 1: Figure S4, where the first 10 panels show the estimated (dashed lines) and true (solid lines) intensity functions in the 10 clusters, and the last panel shows the confusion matrix. We see that, due to the complexity of this scenario, e.g., large number of TFs and randomly assigned clusters, some TFs may be misclassified and the estimation of cluster intensity functions may be biased. For example, it can be seen from Figure S4 that the true clusters 1, 8, and 10 consist of 5, 10, and 7 TFs, respectively, and there exists some similarities in their true intensities. To satisfy the likelihood criterion (2), NLK misclassified 4 TFs from cluster 1 to cluster 10, and 2 TFs from cluster 8 to cluster 1. With only one correct sample path from cluster 1 and two wrong sample paths from cluster 8, numerical searching of the optimal basis coefficient was trapped into a local maxima of the likelihood, resulting in a very biased estimation of the intensity function in cluster 1 (see Panel 1 in Figure S4). However, NLK can still assign most TFs into the correct clusters, as seen from the elements of the main diagonal in the confusion matrix in Figure S4.

### Simulation study to demonstrate NLH clustering

Next, we demonstrate NLH clustering by an additional simulation. The purpose is to see whether the proposed Algorithm 2 is able to uncover the prespecified hierarchy in the simulated NHPP intensities. For this purpose, we consider a simple scenario with $k=3$ clusters formed by $n=10$ TFs, where the three clusters exhibit a hierarchical structure. This scenario, called Scenario 4, adopts a balanced design (3, 3, 4) with the intensity functions of the three clusters set to

$$\begin{gathered} {\text{Cluster 1}}:\lambda _{1} (t) = 5\cos \left( {\frac{{2\pi }}{{10}}t} \right) + 5, \hfill \\ {\text{Cluster 2}}:\lambda _{2} (t) = \frac{5}{2}\cos \left( {\frac{{2\pi }}{{10}}t} \right) + \frac{{15}}{4}\cos \left( {\frac{{4\pi }}{{10}}t} \right) + \frac{{15}}{4}, \hfill \\ {\text{Cluster 3}}:\lambda _{3} (t) = - 5\cos \left( {\frac{{2\pi }}{{10}}t} \right) + 5,\quad t \in [0,10]. \hfill \\ \end{gathered}$$

**Fig. 3:**
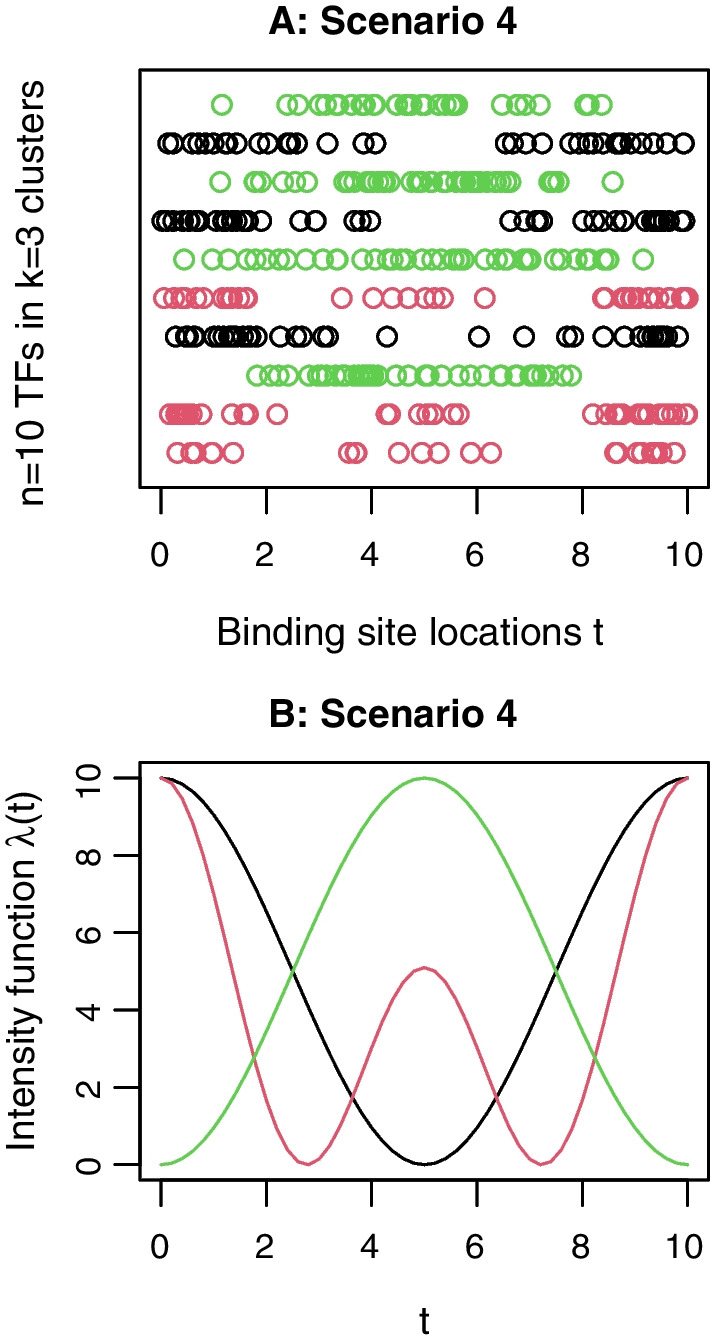
**Demonstration of simulated data in Scenario 4 for NLH clustering.** **A**: Binding site locations of the 10 TFs in Scenario 4, Cluster 1: black, Cluster 2: red, Cluster 3: green; **B**: Intensity functions in the three clusters in Scenario 4, $\lambda _1(t)$: black, $\lambda _2(t)$: red, $\lambda _3(t)$: green

**Fig. 4:**
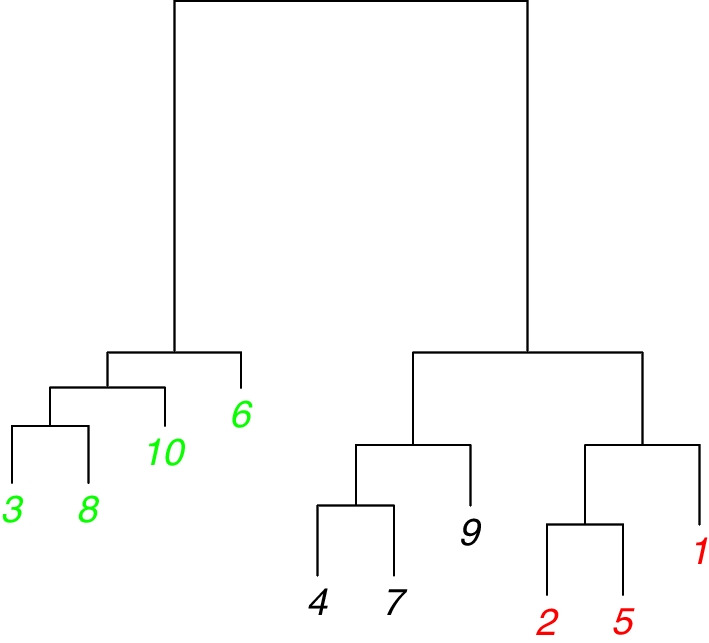
**Demonstration of NLH clustering using example data in Fig.** 3. The tip labels represent the TF IDs, in the order of appearance from bottom to top in Fig. 3**A**. The tip colors indicate the true clusters, Cluster 1: black, Cluster 2: red, Cluster 3: green. The branch lengths represent the between-cluster distances (scaled to [1, 10]) defined by the likelihood linkage

Figure 3 shows the three intensity functions as well as the simulated data for this scenario. It can be seen that, among the three intensity functions, $\lambda _1(t)$ and $\lambda _3(t)$ are farthest in terms of the $L_2$-norm distance as one mirrors the other by flipping vertically. $\lambda _2(t)$ is in the middle but much closer to $\lambda _1(t)$. The three $L_2$-norm distances between $\lambda _1(t)$ and $\lambda _2(t)$, $\lambda _2(t)$ and $\lambda _3(t)$, and $\lambda _1(t)$ and $\lambda _3(t)$ are 10.83, 19.16, and 22.36, respectively. Alternatively, if we apply the likelihood linkage (3) to the simulated clustered data in Fig. 3A, the three empirical between-cluster distances are $D(G_1, G_2)=-26.76, D(G_2, G_3)=262.28$, and $D(G_1, G_3)=333.91$. These distances implies a simple hierarchy of the three clusters: Cluster 1 and Cluster 2 are much less distant thus should be merged first in hierarchical clustering. We apply Algorithm 2 to this set of simulated data, and show the hierarchical clustering result in a dendrogram in Fig. 4. One sees that, NLH clustering is able to find the correct clusters for this example data as samples within each cluster exhibit markedly shorter distances. Moreover, after the three clusters (shown in green, black, and red) are found one by one, they start to merge into clades until the root appears. Clusters 1 (black) and 2 (red) merge before Cluster 3 (green) joins according to our calculation on the between-cluster distances defined by the likelihood linkage. These results justify the effectiveness of NLH clustering.

### Application to ChIP-seq data generated from mouse ESCs

**Fig. 5:**
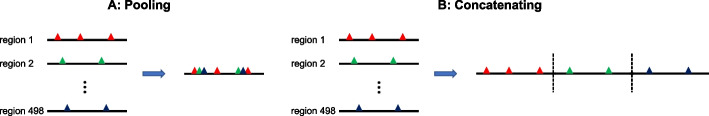
**A schematic plot to illustrate the difference between the pooling and concatenating strategies.** The target genomic region is defined by **A**: pooling together the BS locations of each TF on the upstream [−8K, 2K] region of different genes; **B**: concatenating the upstream [−8K, 2K] region of different genes

In the real data analysis, we use multiple-TF ChIP-seq data collected in recent mouse embryonic stem cell research [10, 18, 19] and apply the proposed NHPP-likelihood-based clustering methods to identify possible TF clusters. The TFs under consideration include Esrrb, Nanog, Oct4, Sox2, E2f1, Smad1, Tcfcp2l1, Zfx, Klf4, cMyc, nMyc, Stat3, Nr5a2, and Tcf3. As mentioned in Methods, different analyses can be performed depending on how the target genomic region is chosen. Here we focus on the upstream gene regions as done in Cha and Zhou [15]. The preprocessing includes a few steps: First, for each of the 14 TFs, its BS locations on the upstream [−8K, 2K] region relative to the gene TSS were retrieved for a total of 28,353 genes on the mouse genome. Second, to study TF co-localization, the 28,353 genes were filtered by thresholding the number of TFs binding to their upstream regions. Here we focused on genes on which more than 50% (i.e., $\ge 8$) TFs have BSs, and retained 498 “informative” genes. Third, two different strategies, “pooling” and “concatenating”, were adopted for constructing the target genomic region: (1) We may pool together the BS locations of each TF on the 498 upstream gene regions, by assuming that the binding intensity of the TF is identical across all regions [15]. (2) Alternatively, if the identical binding intensity assumption cannot be satisfied (which seems to be more realistic for practical use), we may simply concatenate the 498 upstream gene regions to define the target genomic region. A schematic plot is shown in Fig. 5 to illustrate the difference between the pooling and concatenating strategies. Finally, the target genomic region was mapped to a prescribed interval, for example [0, 10], for subsequent clustering analysis. The BS locations were then scaled correspondingly. Table 3 and Additional file 1: Table S1 report the number of BS locations of the 14 TFs together with the 6-number summary statistics of the scaled BS locations, on a target genomic region constructed by using the pooling and concatenating strategies, respectively.

**Table 3:** Summary of BS locations for 14 TFs on 498 upstream gene regions in real application, using the pooling strategy

TF	No. of BS	Summary of BS locations$^*$					
		Min.	1st Qu.	Median	Mean	3rd Qu.	Max.
Esrrb	698	0.026	2.707	5.807	5.287	7.833	9.983
Nanog	414	0.004	2.372	5.055	4.893	7.399	9.955
Oct4	367	0.030	2.744	6.490	5.407	7.859	9.969
Sox2	288	0.072	2.308	4.866	4.841	7.432	9.929
E2f1	1,286	0.002	3.628	6.692	5.813	8.025	9.951
Smad1	123	0.020	1.853	4.911	4.720	7.020	9.938
Tcfcp2l1	744	0.003	3.504	6.382	5.622	7.934	9.991
Zfx	575	0.013	4.023	7.332	5.984	8.058	9.937
Klf4	610	0.034	3.615	7.188	5.919	7.940	9.850
cMyc	370	0.011	5.700	7.659	6.557	8.158	9.962
nMyc	567	0.044	4.595	7.569	6.288	8.027	9.993
Stat3	255	0.040	2.838	5.561	5.133	7.636	9.911
Nr5a2	36	0.680	1.964	4.752	4.425	6.820	8.820
Tcf3	248	0.014	2.459	4.744	4.942	7.070	9.911

$^*$ These BS locations were scaled to [0, 10]

**Fig. 6:**
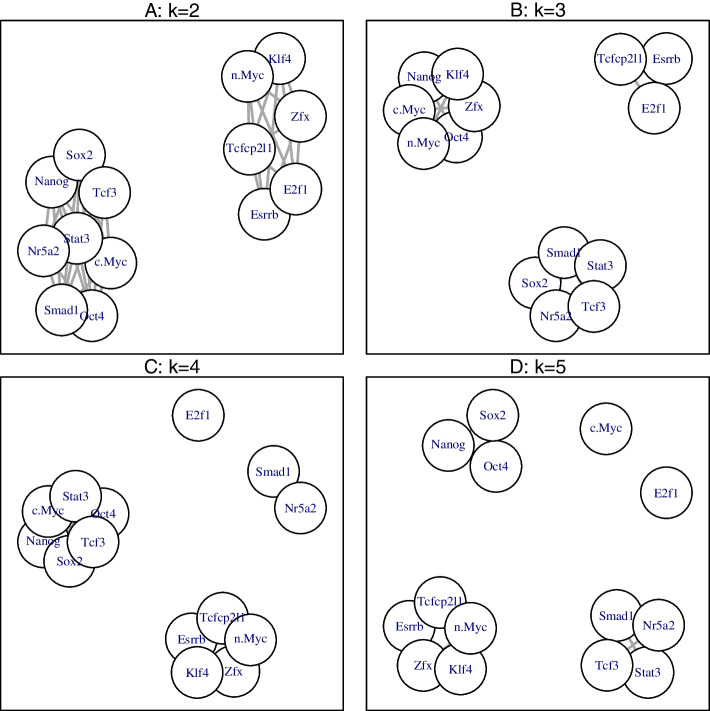
NLK clustering of 14 TFs in real application using pooling strategy, for different number of clusters $k=2, 3, 4$, and 5

The NLK clustering results using the pooling strategy are shown in Fig. 6 for different number of clusters $k=2, 3, 4$, and 5. As a comparison, Additional file 1: Figure S5 shows the NLK clustering results using the concatenating strategy. Clearly, the two strategies provide similar (for $k=4$ and $k=5$) or even identical (for $k=2$ and $k=3$) clustering results. In particular, when $k=2$, NLK detects two clusters, one includes Nanog, Oct4, Sox2, Smad1, Stat3, etc., and the other consists of cMyc, nMyc, Zfx, E2f1, etc. This finding is consistent with that in previous studies [10, 15, 20]. It can also be seen from Fig. 6 that, as the number of clusters increases from $k=4$ to $k=5$, the cluster of Nanog, Oct4, and Sox2 stands out gradually. In the literature, Nanog, Oct4, and Sox2 are known as essential TFs to maintain the pluripotent ESC phenotype [21, 22]. It has been found that the Nanog-Oct4-Sox2 cluster exhibits features of enhanceosomes [10] and the three TFs collaborate to maintain pluripotency [23] and form transcriptional regulatory circuitry [24].

**Fig. 7:**
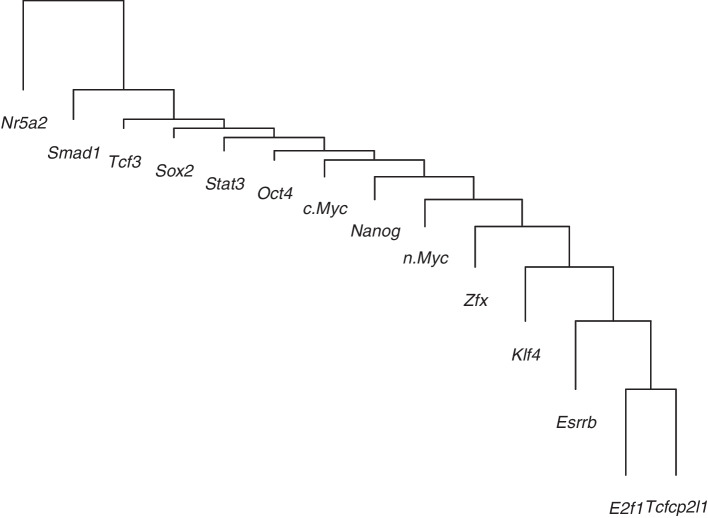
**NLH clustering dendrogram of 14 TFs in real application using pooling strategy.** The branch lengths represent the between-cluster distances (scaled to [1, 10]) defined by the likelihood linkage. From bottom to top, the scaled branch lengths are: 9.60, 7.62, 6.05, 4.54, 3.02 (median), 2.54, 1.88, 1.05, 1.46, 1.04, 1.00, 3.30, 10.00

Figure 7 and Additional file 1: Figure S6 show the NLH clustering dendrograms using the pooling and concatenating strategies, respectively. Again, we see that the two strategies yield quite similar NLH clustering results. The two dendrograms only have minor differences in the order of merging Sox2, Stat3, Oct4, and cMyc. It is worth noting that, since the branch lengths represent the between-cluster distances defined by the likelihood linkage, shorter branch lengths (or higher NHPP-likelihoods) indicate the formation of tighter clusters. In the two dendrograms, if we only focus on the shorter branch lengths, e.g., those smaller than the median (the branch length of nMyc), then the corresponding TFs in merge are Nanog, cMyc, Oct4, Stat3, Sox2, and Tcf3. This suggests closer cooperation of these TFs in producing combinatorial binding patterns. Besides the three core ESC regulaors, Nanog, Oct4, and Sox2, it has been found that Tcf3 shares highly overlapping occupancy with Oct4, Sox2, and Nanog at the TSS of miRNA transcripts [19]. cMyc is one of the “four factors” that are sufficient to reprogram mouse fibroblasts to induced pluripotent stem (iPS) cells [25]. When expressed individually in fibroblasts, cMyc promotes the most ES cell-like transcription pattern [26]. Stat3, together with cMyc, plays an important role in regulating pluripotency-related gene expression in ESCs [27]. These findings are supported by our clustering analyses using the proposed methods.

## Discussion

In this paper, we present a novel statistical framework for detecting clusters of transcription factor binding patterns. We modeled TF binding events by nonhomogeneous Poisson processes and developed partitional and hierarchical clustering methods, NLK and NLH, based on the NHPP likelihood. Simulation studies showed that, the NLK clustering generally achieved more accurate results than the traditional window-based K-means clustering; the NLH clustering successfully uncovered the hierarchy in the NHPP intensities. The proposed clustering methods were then applied to multiple-TF ChIP-seq data in mouse embryonic stem cells. The application provided not only evidences to support the previously identified cluster of core ESC regulators but also new insights on functional implications of transcrisptional regulatory modules.

The goal of this work is to provide a creative and original tool for geneticists and bioinformaticians to extract meaningful information out of the wealth modern omics data, thereby advancing our understanding of gene regulatory mechanisms. We note that, the current study is limited to the analysis of ChIP-seq data only, thus may not derive full information of the complicated process of transcriptional regulation. In the future, an integrated analysis of omics data (e.g., ChIP-seq, RNA-seq, and methylome) will be desirable to help us better understand the orchestration of transcription factors and other proteins during complex biological processes.

It is noteworthy that, we adopted the clustering methodology to investigate how TFs with similar binding patterns may work together. In contrast, there is also sophisticated methodology for studying the interactions among TFs, such as whether and how the presence of certain TFs may trigger other TFs’ binding behavior to regulate the expression of target genes. Though the two methodologies could be developed under the same NHPP model, they have markedly differences on the goals and approaches, and should not be misused. Statistically, the former relies on similarity/distance metrics among TF binding patterns, whereas the latter tries to address the (conditional) dependence relationship among TFs on the transcriptional circuitry. An interesting future direction following our NHPP framework may be to develop state-of-the-art statistical methods, such as Gaussian process graphical models [28, 29], to decipher the transcriptional regulatory networks.

Occasionally, the pairwise relation between two TF binding patterns may be of main interest. For example, Oct4 and Sox2 have been shown to bind cooperatively to two adjacent *cis*-regulatory elements located within a powerful enhancer of the FGF4 gene [30]. The problem of detecting pairwise TF relation can usually be addressed by hypothesis testing. An example can be found in Cha and Zhou, where an asymptotic Z test is proposed to test independent binding of two TFs based on NHPP model and Ripley’s K-function [15]. We note that, hypothesis testing is also applicable under our NHPP modeling framework. For example, a likelihood ratio test (LRT) can be easily derived to check whether two TFs share the same binding pattern on the target genomic region, that is, to decide between $H_0: \lambda _1(t)=\lambda _2(t)$ and $H_a: \lambda _1(t)\ne \lambda _2(t)$ for $t\in D$, where $\lambda _1(t)$ and $\lambda _2(t)$ are the binding intensity functions of the two TFs. It is, however, generally not appropriate to use pairwise relation analysis (with heatmap output) to solve the problem of multiple TF clustering (with dendrogram output). As seen in the real data analysis of Cha and Zhou [15], among the total 91 TF pairs, 86 were found to have significant clustering patterns with an FDR 52%, showing the excessive false positive rate (i.e., almost all TF pairs exhibit clustering/repulsive pattern) of their hypothesis testing method.

Estimation of the NHPP intensity function is the cornerstone of our developed NHPP-likelihood-based clustering methods. As presented in Methods, we focus on maximum likelihood estimation of the NHPP intensity function using basis expansion, particularly DCT. Various methods for estimating the NHPP intensity function have been developed in the literature, including wavelet-based methods [31–33], and kernel-based methods [34–36]. In practice, one may choose a suitable method with a balance on estimation accuracy and computational efficiency.

Another concern specifically on the NLH clustering is the linkage. Besides the proposed NHPP-likelihood linkage (3), other similarity or distance metrics between two probability distributions may also be applicable, e.g., the Hellinger distance, the Bhattacharyya distance, and the Kullback-Leibler divergence [37, 38]. It is worth exploring some NLH variations by treating these alternatives as the distance between two clusters in the context of NHPP, i.e., between two NHPP intensity functions.

## Supplementary Information

**Additional file 1.** Supplementary Figures S1–S6 and Table S1.

## Data Availability

All data analyzed in this study are publicly available in Chen et al. [10], Heng et al. [18], and Marson et al. [19].

## Abbreviations

TF: Transcription factor
BS: Binding site
ChIP-seq: Chromatin immunoprecipitation combined with sequencing
ESCs: Embryonic stem cells
TSS: Transcription start site
NHPPs: Nonhomogeneous Poisson processes
NLK: NHPP-likelihood-based K-means
NLH: NHPP-likelihood-based hierarchical-clustering
DCT: Discrete cosine transform
AMCR: Average misclassification rate
PPC: Proportion of perfect classification
LRT: Likelihood ratio test
