# Supplementary material for: Detecting clusters of transcription factors based on a nonhomogeneous poisson process model
Source: BMC Bioinformatics. 2022 Dec 9;23:535. doi: 10.1186/s12859-022-05090-2 (PMC9738027; doi:10.1186/s12859-022-05090-2)

Supplementary Materials for "Detecting Clusters of Transcription Factors based on a Nonhomogeneous Poisson Process Model"

Xiaowei Wu[1*], Shicheng Liu[2], and Guanying Liang[2]

[1]Department of Statistics, Virginia Tech, 250 Drillfield Drive, Blacksburg, VA 24061, USA

[2]Department of Mathematics, Virginia Tech, 225 Stanger Street, Blacksburg, VA 24061, USA

[*]Corresponding author

Email: xwwu@vt.edu

Figure S1: **Demonstration of simulated data in Scenarios 3.** A: Binding site locations of the 100 TFs in Scenario 3; B: Intensity functions in the 10 clusters in Scenario 3. The 10 clusters are displayed in different colors.

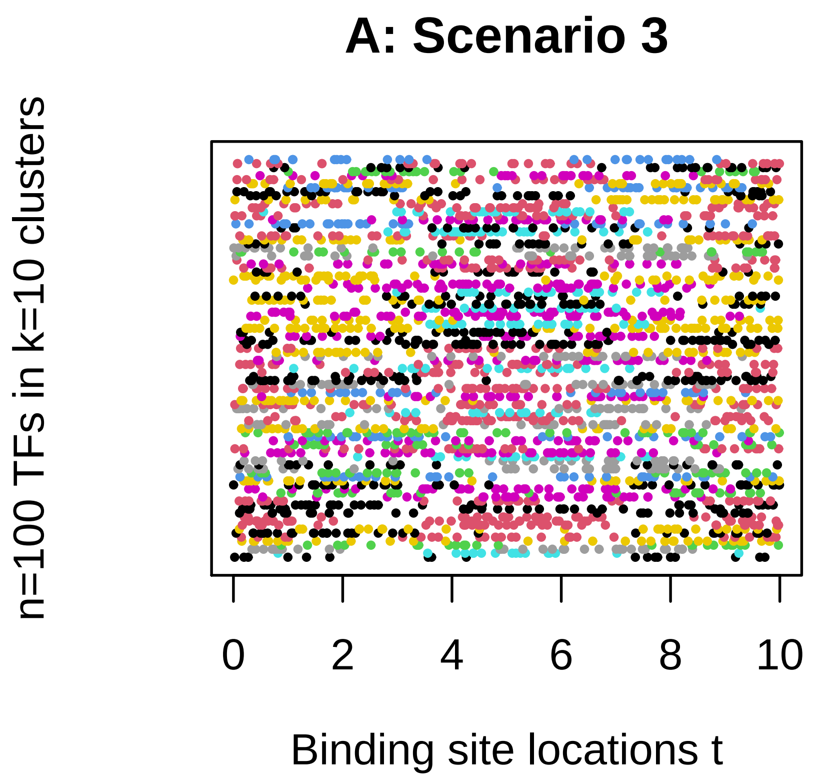

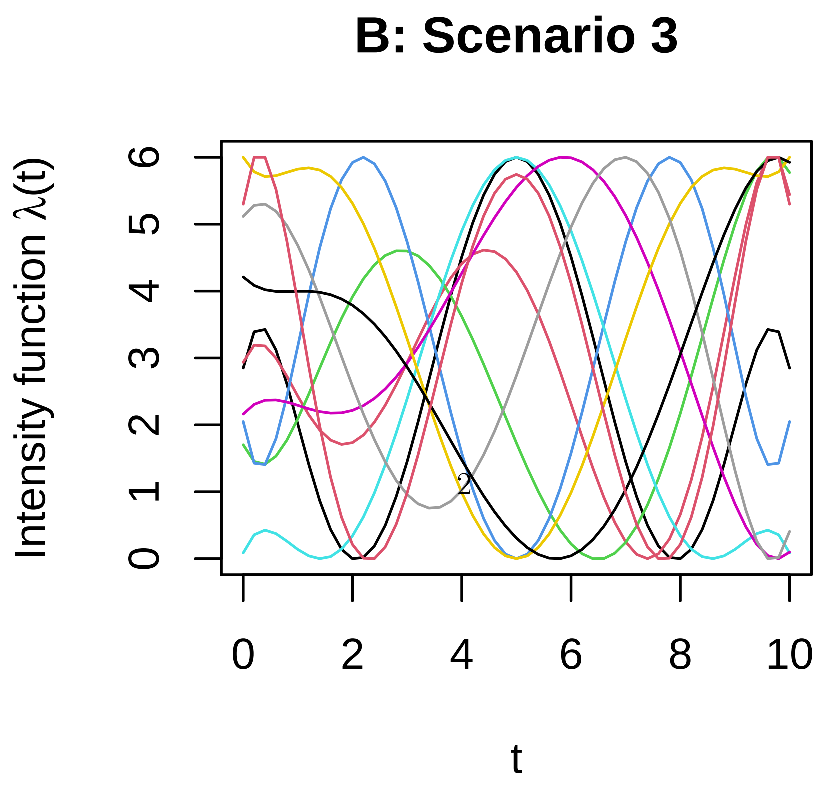

Figure S2: **Relation between the NLK clustering performance and the number of TFs included.** A: AMCR decreases as the number of TFs increases; B: PPC decreases gradually as the number of TFs increases.

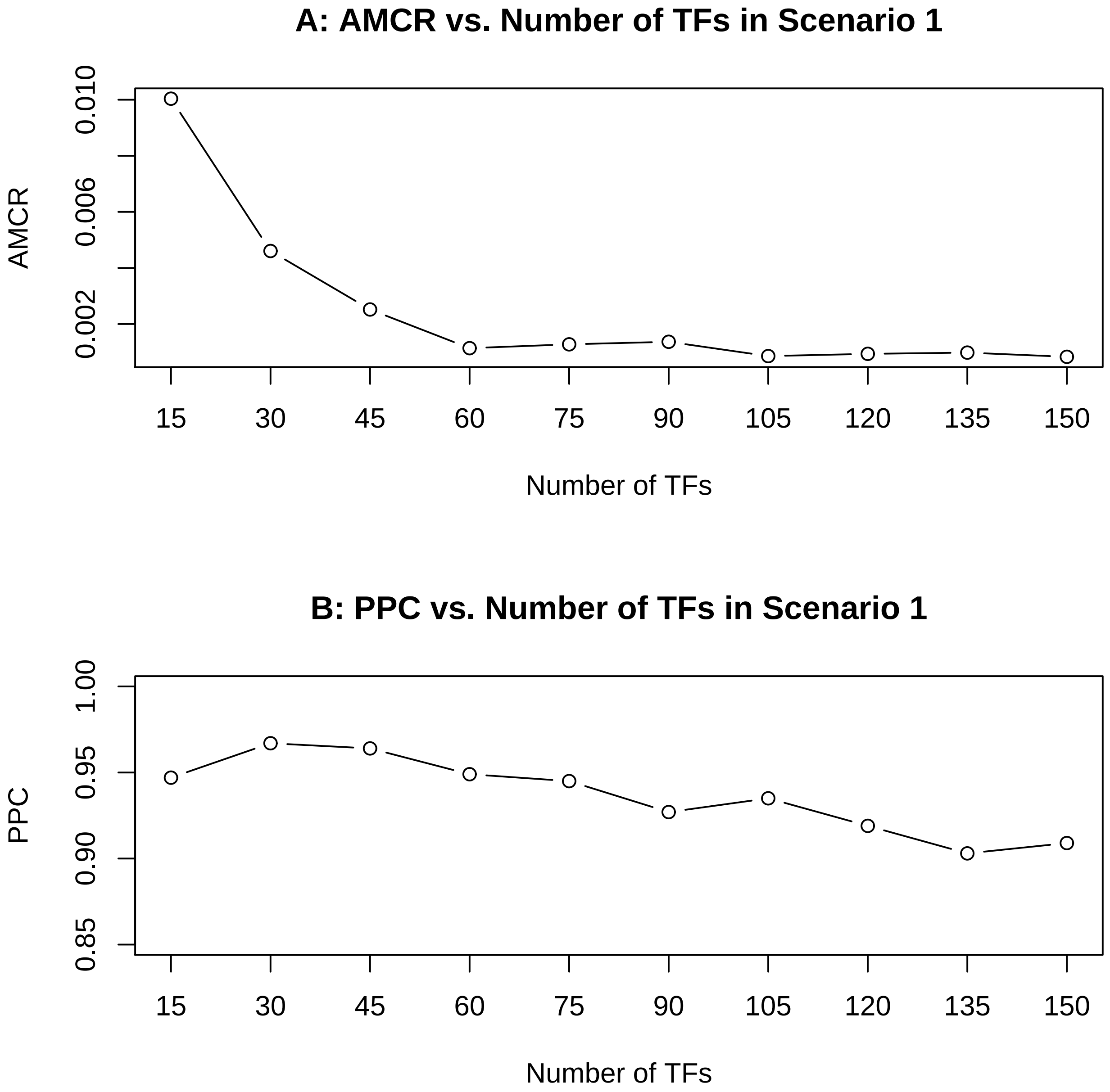

Figure S3: **Comparison of average misclassification rates (AMCR) using simulated data in Scenarios 1∼3.** A: Scenario 1; B: Scenario 2; C: Scenario 3. For each scenario, the AMCR of window-based K-means under different window widths is displayed in black curve, and the AMCR of NHPP-based K-means is displayed in horizontal red line.

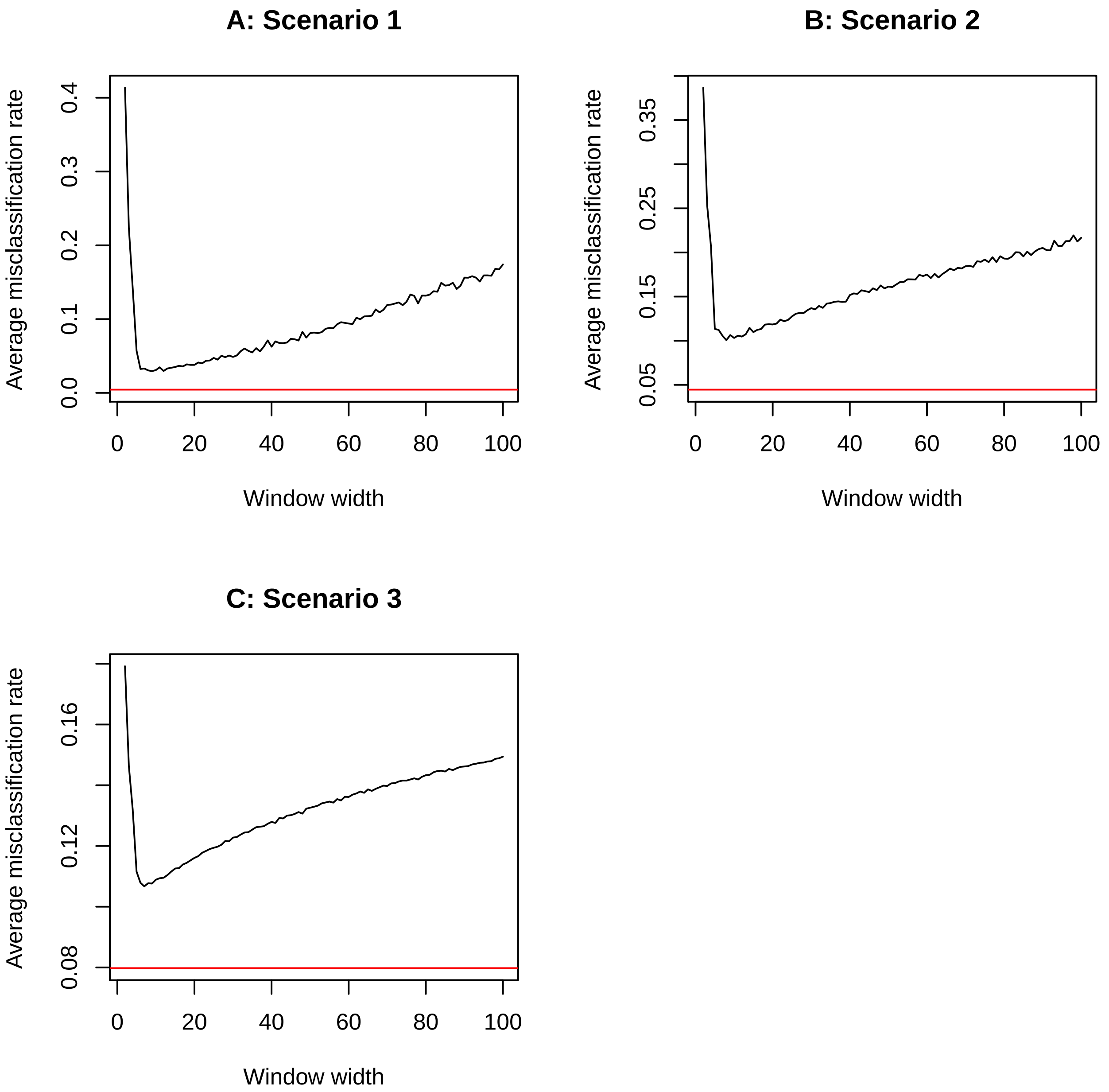

Figure S4: **Demonstration of intensity function estimation using simulated data in Scenario 3.** The first 10 panels show the true (solid lines) and estimated (dashed lines) intensity functions in the 10 clusters. The last panel shows the confusion matrix.

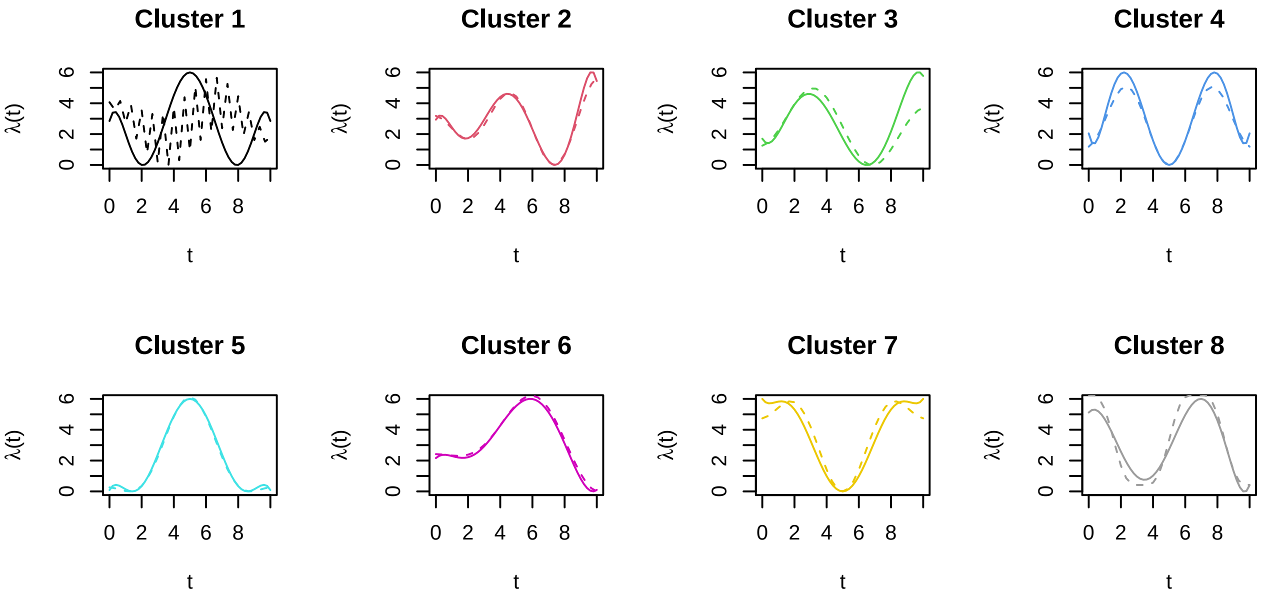

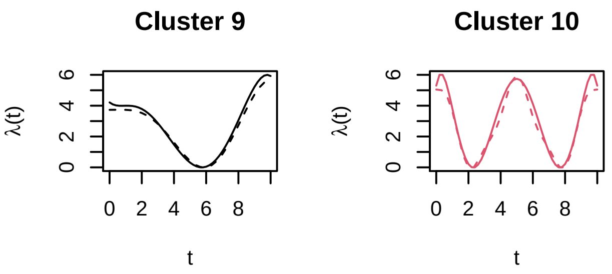

Estimated Class

True Class	1	2	3	4	5	6	7	8	9	10
1	1	0	0	0	0	0	0	0	0	4
2	0	13	0	0	0	0	0	0	0	0
3	0	0	9	0	0	0	0	0	0	0
4	0	0	0	10	0	0	2	0	0	0
5	0	0	0	0	9	0	0	0	0	0
6	0	0	0	0	0	10	0	0	0	0
7	0	0	0	0	0	0	12	0	0	0
8	2	0	0	0	0	0	0	8	0	0
9	0	0	0	0	0	0	0	0	12	0
10	0	0	0	0	0	0	0	0	0	7

Table S1: **Summary of BS locations for 14 TFs on 498 upstream gene regions in real application, using the concatenating strategy.**

TF	No. of BS	Summary of BS locations*					
		Min.	1st Qu.	Median	Mean	3rd Qu.	Max.
Esrrb	698	0.009	2.248	5.033	4.971	7.438	9.994
Nanog	414	0.010	2.411	4.875	4.846	7.193	9.987
Oct4	367	0.052	2.772	4.951	5.066	7.468	9.988
Sox2	288	0.104	2.677	4.793	4.881	7.225	9.988
E2f1	1,286	0.006	1.961	4.597	4.736	7.359	9.996
Smad1	123	0.052	2.260	4.271	4.313	6.184	9.953
Tcfcp2l1	744	0.006	2.463	5.047	5.061	7.715	10.00
Zfx	575	0.017	2.513	5.198	5.134	7.918	9.927
Klf4	610	0.009	2.300	4.933	4.974	7.647	9.928
cMyc	370	0.077	2.150	4.807	5.025	7.807	9.936
nMyc	567	0.015	2.475	5.136	5.091	7.726	9.996
Stat3	255	0.009	2.690	5.937	5.556	8.474	9.953
Nr5a2	36	0.336	1.705	4.174	4.240	6.970	8.106
Tcf3	248	0.009	2.646	5.180	4.930	6.943	9.994

* These BS locations were scaled to $[0, 10]$.

Figure S5: **NLK clustering of 14 TFs in real application using concatenating strategy, for different number of clusters $k = 2, 3, 4$, and 5.**

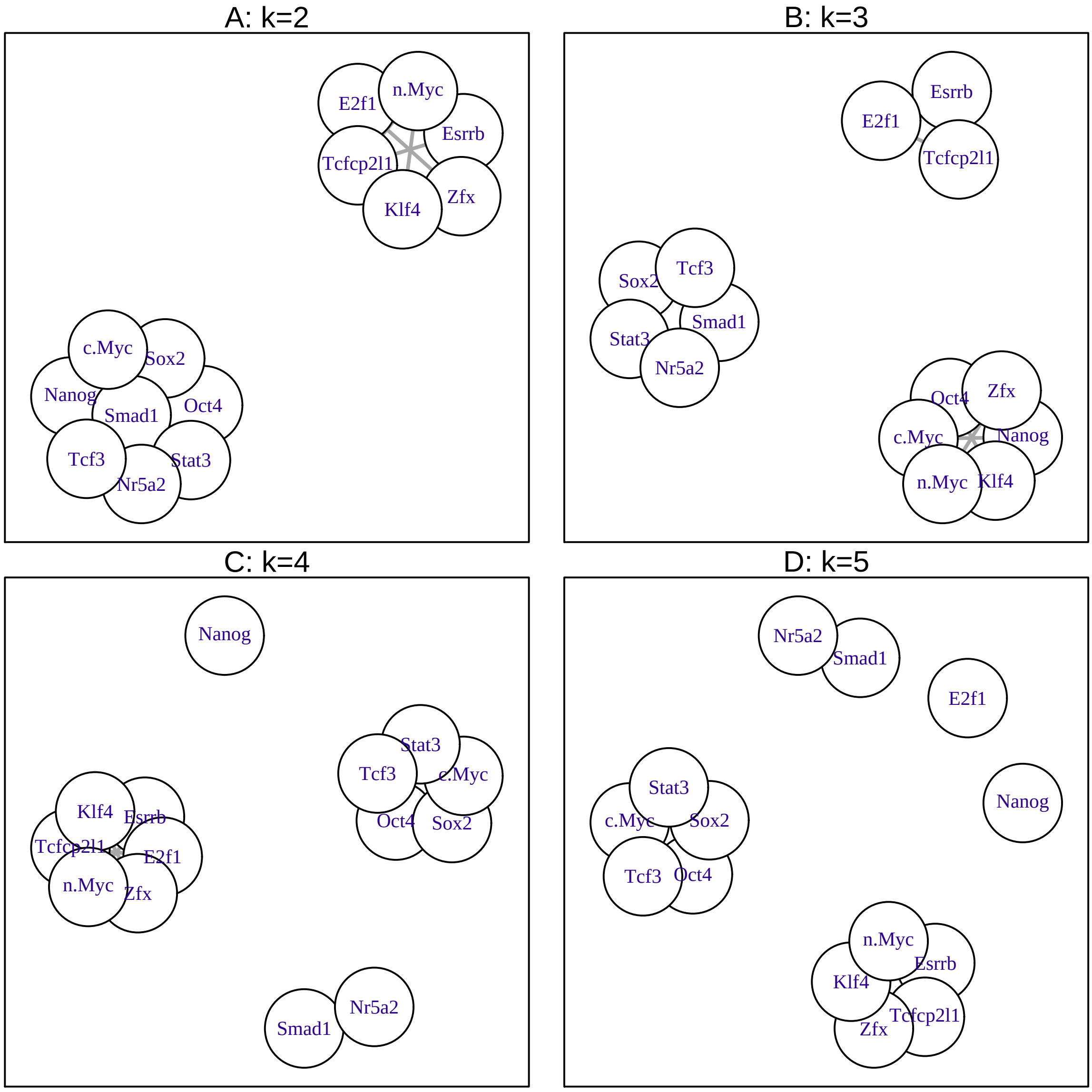

Figure S6: **NLH clustering dendrogram of 14 TFs in real application using concatenating strategy.** The branch lengths represent the between-cluster distances (scaled to [1, 10]) defined by the likelihood linkage. From bottom to top, the scaled branch lengths are: 9.14, 7.30, 5.86, 4.49, 3.13 (median), 2.73, 2.12, 1.34, 1.39, 1.30, 1.00, 3.59, 10.00.

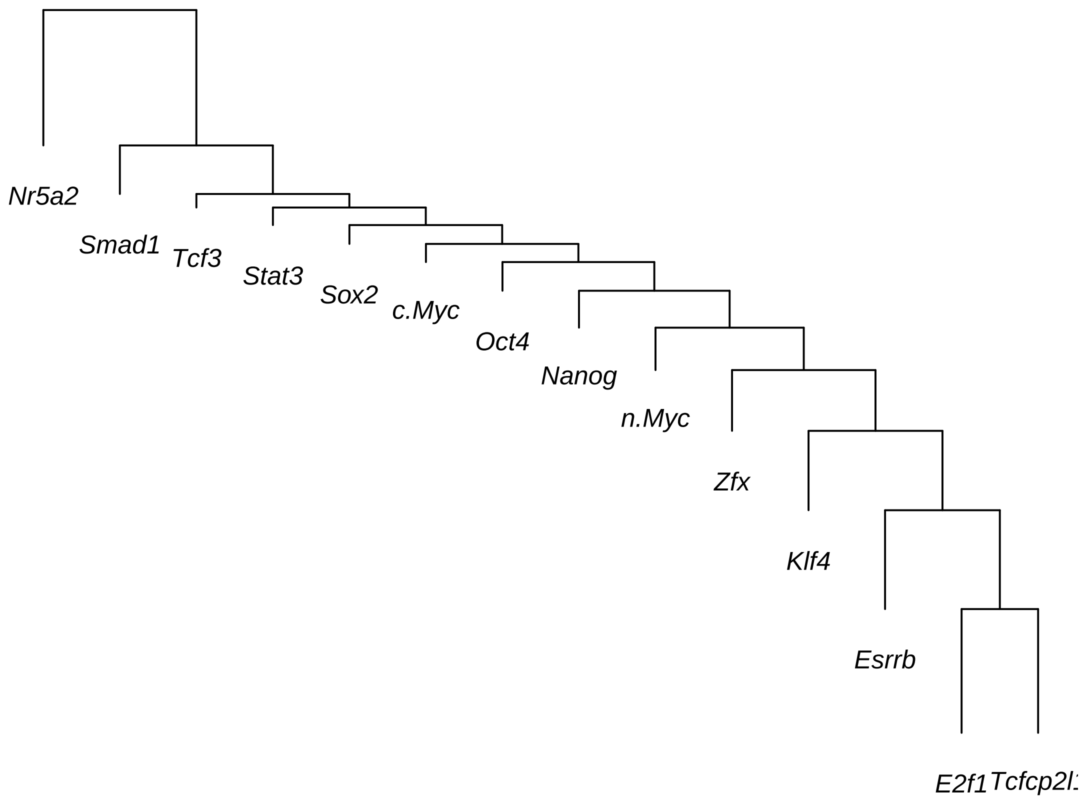

Supplement: Supplementary file 1 — Additional file 1. Supplementary Figures S1–S6 and Table S1. [file 12859_2022_5090_MOESM1_ESM.pdf]
